# Supplementary material for: Multi‐dataset identification of innovative feature genes and molecular mechanisms in keratoconus
Source: J Cell Mol Med. 2024 Sep 19;28(18):e70079. doi: 10.1111/jcmm.70079 (PMC11412914; doi:10.1111/jcmm.70079)
Supplement: Supplementary file 2 — Material S2. [file JCMM-28-e70079-s002.zip › 7.GSEA analysis/4.MYRF/KEGG_TOLL_LIKE_RECEPTOR_SIGNALING_PATHWAY.html]

Details for gene set KEGG\_TOLL\_LIKE\_RECEPTOR\_SIGNALING\_PATHWAY[GSEA]

|  || Dataset | MYRF.group.cls#h\_versus\_l |
| Phenotype | group.cls#h\_versus\_l |
| Upregulated in class | l |
| GeneSet | KEGG\_TOLL\_LIKE\_RECEPTOR\_SIGNALING\_PATHWAY |
| Enrichment Score (ES) | -0.44672948 |
| Normalized Enrichment Score (NES) | -1.549126 |
| Nominal p-value | 0.016701462 |
| FDR q-value | 0.20193149 |
| FWER p-Value | 0.689 |
Table: GSEA Results Summary

  

Fig 1: Enrichment plot: KEGG\_TOLL\_LIKE\_RECEPTOR\_SIGNALING\_PATHWAY      
 Profile of the Running ES Score & Positions of GeneSet Members on the Rank Ordered List

  

| SYMBOL | TITLE | RANK IN GENE LIST | RANK METRIC SCORE | RUNNING ES | CORE ENRICHMENT || 1 | CD86 | na | 355 | 0.626 | 0.0221 | No |
| 2 | IFNA14 | na | 451 | 0.586 | 0.0497 | No |
| 3 | PIK3R5 | na | 661 | 0.528 | 0.0710 | No |
| 4 | IL12A | na | 1851 | 0.369 | 0.0558 | No |
| 5 | IFNA6 | na | 2154 | 0.344 | 0.0649 | No |
| 6 | IFNA4 | na | 3565 | 0.269 | 0.0381 | No |
| 7 | TLR8 | na | 4025 | 0.253 | 0.0380 | No |
| 8 | IRF5 | na | 4432 | 0.240 | 0.0387 | No |
| 9 | IFNA16 | na | 6382 | 0.181 | -0.0082 | No |
| 10 | PIK3R1 | na | 7105 | 0.161 | -0.0208 | No |
| 11 | TIRAP | na | 7289 | 0.156 | -0.0180 | No |
| 12 | MAPK9 | na | 8409 | 0.129 | -0.0436 | No |
| 13 | MAPK12 | na | 8572 | 0.125 | -0.0418 | No |
| 14 | CASP8 | na | 9334 | 0.109 | -0.0581 | No |
| 15 | RIPK1 | na | 9519 | 0.105 | -0.0580 | No |
| 16 | PIK3CG | na | 9794 | 0.100 | -0.0607 | No |
| 17 | IRAK1 | na | 10013 | 0.096 | -0.0621 | No |
| 18 | TRAF3 | na | 10032 | 0.095 | -0.0577 | No |
| 19 | AKT2 | na | 10210 | 0.092 | -0.0580 | No |
| 20 | MAPK11 | na | 10606 | 0.085 | -0.0650 | No |
| 21 | TOLLIP | na | 10634 | 0.084 | -0.0615 | No |
| 22 | FADD | na | 10767 | 0.082 | -0.0610 | No |
| 23 | TLR5 | na | 10829 | 0.081 | -0.0586 | No |
| 24 | MAPK13 | na | 10951 | 0.079 | -0.0580 | No |
| 25 | PIK3CA | na | 11255 | 0.074 | -0.0629 | No |
| 26 | MAPK14 | na | 11965 | 0.064 | -0.0801 | No |
| 27 | SPP1 | na | 12214 | 0.060 | -0.0841 | No |
| 28 | CD14 | na | 12315 | 0.059 | -0.0840 | No |
| 29 | MAPK10 | na | 12440 | 0.057 | -0.0846 | No |
| 30 | PIK3R2 | na | 12535 | 0.056 | -0.0844 | No |
| 31 | STAT1 | na | 12606 | 0.055 | -0.0835 | No |
| 32 | IKBKB | na | 12994 | 0.050 | -0.0921 | No |
| 33 | MAP2K4 | na | 13264 | 0.047 | -0.0975 | No |
| 34 | MAP2K2 | na | 14012 | 0.038 | -0.1171 | No |
| 35 | CHUK | na | 14348 | 0.034 | -0.1250 | No |
| 36 | MAPK3 | na | 14382 | 0.034 | -0.1242 | No |
| 37 | TLR3 | na | 14431 | 0.033 | -0.1239 | No |
| 38 | IFNA2 | na | 14988 | 0.028 | -0.1385 | No |
| 39 | JUN | na | 16671 | 0.011 | -0.1865 | No |
| 40 | PIK3CB | na | 17199 | 0.007 | -0.2013 | No |
| 41 | IFNB1 | na | 17252 | 0.006 | -0.2025 | No |
| 42 | AKT1 | na | 18101 | -0.001 | -0.2269 | No |
| 43 | MAPK8 | na | 18612 | -0.006 | -0.2413 | No |
| 44 | TAB1 | na | 19282 | -0.014 | -0.2599 | No |
| 45 | MAP2K7 | na | 19405 | -0.015 | -0.2626 | No |
| 46 | IFNAR1 | na | 19408 | -0.015 | -0.2619 | No |
| 47 | IRF7 | na | 19740 | -0.019 | -0.2705 | No |
| 48 | MYD88 | na | 20189 | -0.024 | -0.2822 | No |
| 49 | MAPK1 | na | 20406 | -0.027 | -0.2871 | No |
| 50 | PIK3CD | na | 20433 | -0.027 | -0.2864 | No |
| 51 | PIK3R3 | na | 20464 | -0.027 | -0.2859 | No |
| 52 | IRAK4 | na | 20511 | -0.028 | -0.2858 | No |
| 53 | FOS | na | 20589 | -0.029 | -0.2865 | No |
| 54 | TBK1 | na | 21332 | -0.037 | -0.3060 | No |
| 55 | IKBKG | na | 21687 | -0.042 | -0.3141 | No |
| 56 | RAC1 | na | 21936 | -0.045 | -0.3189 | No |
| 57 | TAB2 | na | 22545 | -0.053 | -0.3337 | No |
| 58 | IL12B | na | 22624 | -0.054 | -0.3332 | No |
| 59 | TLR1 | na | 23500 | -0.068 | -0.3550 | No |
| 60 | TRAF6 | na | 23526 | -0.068 | -0.3522 | No |
| 61 | MAP2K3 | na | 23613 | -0.069 | -0.3511 | No |
| 62 | CTSK | na | 24373 | -0.082 | -0.3687 | No |
| 63 | IRF3 | na | 24400 | -0.082 | -0.3652 | No |
| 64 | MAP3K7 | na | 24724 | -0.088 | -0.3700 | No |
| 65 | CD80 | na | 26452 | -0.122 | -0.4135 | No |
| 66 | IKBKE | na | 26496 | -0.123 | -0.4084 | No |
| 67 | IFNAR2 | na | 27532 | -0.149 | -0.4306 | No |
| 68 | LBP | na | 27766 | -0.154 | -0.4293 | No |
| 69 | TLR7 | na | 28159 | -0.165 | -0.4321 | No |
| 70 | NFKB1 | na | 28241 | -0.168 | -0.4258 | No |
| 71 | CXCL11 | na | 28291 | -0.170 | -0.4184 | No |
| 72 | IFNA7 | na | 28684 | -0.181 | -0.4204 | No |
| 73 | IFNA8 | na | 29598 | -0.210 | -0.4359 | Yes |
| 74 | TLR2 | na | 29913 | -0.220 | -0.4335 | Yes |
| 75 | MAP2K1 | na | 30150 | -0.229 | -0.4285 | Yes |
| 76 | MAP2K6 | na | 30203 | -0.231 | -0.4181 | Yes |
| 77 | CCL5 | na | 31086 | -0.268 | -0.4296 | Yes |
| 78 | RELA | na | 31135 | -0.271 | -0.4170 | Yes |
| 79 | LY96 | na | 31218 | -0.276 | -0.4051 | Yes |
| 80 | NFKBIA | na | 31525 | -0.292 | -0.3989 | Yes |
| 81 | TLR4 | na | 31882 | -0.311 | -0.3931 | Yes |
| 82 | TLR6 | na | 32110 | -0.324 | -0.3829 | Yes |
| 83 | IFNA10 | na | 32142 | -0.326 | -0.3669 | Yes |
| 84 | CD40 | na | 32572 | -0.356 | -0.3609 | Yes |
| 85 | TICAM1 | na | 32573 | -0.356 | -0.3424 | Yes |
| 86 | IL6 | na | 33353 | -0.425 | -0.3430 | Yes |
| 87 | IFNA21 | na | 34032 | -0.529 | -0.3352 | Yes |
| 88 | TLR9 | na | 34231 | -0.580 | -0.3109 | Yes |
| 89 | IL1B | na | 34248 | -0.586 | -0.2811 | Yes |
| 90 | TNF | na | 34328 | -0.609 | -0.2518 | Yes |
| 91 | MAP3K8 | na | 34409 | -0.639 | -0.2211 | Yes |
| 92 | CCL3 | na | 34424 | -0.643 | -0.1882 | Yes |
| 93 | CXCL8 | na | 34657 | -0.838 | -0.1516 | Yes |
| 94 | CXCL9 | na | 34681 | -0.884 | -0.1065 | Yes |
| 95 | AKT3 | na | 34696 | -0.932 | -0.0587 | Yes |
| 96 | CXCL10 | na | 34719 | -1.151 | 0.0003 | Yes |
Table: GSEA details [plain text format]

  

Fig 2: KEGG\_TOLL\_LIKE\_RECEPTOR\_SIGNALING\_PATHWAY      
 Blue-Pink O' Gram in the Space of the Analyzed GeneSet

  

Fig 3: KEGG\_TOLL\_LIKE\_RECEPTOR\_SIGNALING\_PATHWAY: Random ES distribution      
 Gene set null distribution of ES for **KEGG\_TOLL\_LIKE\_RECEPTOR\_SIGNALING\_PATHWAY**
